# High SHIP2 Expression Indicates Poor Survival in Colorectal Cancer

**DOI:** 10.1155/2014/218968

**Published:** 2014-11-24

**Authors:** Ju Yang, Maoying Fu, Yaoguang Ding, Yajing Weng, Weifei Fan, Xiaolin Pu, Zhijun Ge, Feng Zhan, Huihui Ni, Wei Zhang, Feng Jin, Ning Xu, Jiang Li, Liang Qiu, Jun Wang, Xuefeng Gu

**Affiliations:** ^1^Department of Gastroenterology, Kunshan Hospital of Traditional Chinese Medicine, The Affiliated Hospital of Nanjing University of Chinese Medicine, Suzhou 215000, China; ^2^Department of Infectious Diseases, The First People's Hospital of Kunshan Affiliated with Jiangsu University, Suzhou 215000, China; ^3^Department of Oncology, Kunshan Hospital of Traditional Chinese Medicine, The Affiliated Hospital of Nanjing University of Chinese Medicine, Suzhou 215000, China; ^4^Department of Geriatric Rehabilitation, Kunshan Hospital of Traditional Chinese Medicine, The Affiliated Hospital of Nanjing University of Chinese Medicine, Suzhou 215000, China; ^5^Department of Hematology and Oncology, Jiangsu Province Geriatric Institute, Nanjing 210000, China; ^6^Department of Anesthesiology, Yixing People's Hospital, The Affiliated Yixing Hospital of Jiangsu University, Yixing 214200, China; ^7^Department of Hepatobiliary and Laparoscopic Surgery, Yixing People's Hospital, The Affiliated Yixing Hospital of Jiangsu University, Yixing 214200, China; ^8^Department of Pathology, Nanjing Medical University, Nanjing 210000, China; ^9^Department of Pathology, Jiangsu Province Geriatric Institute, Nanjing 210000, China

## Abstract

SH2-containing inositol 5′-phosphatase 2 (SHIP2), which generally regulates insulin signaling, cytoskeleton remodeling, and receptor endocytosis, has been suggested to play a significant role in tumor development and progression. However, the associations between SHIP2 expression and the clinical features to evaluate its clinicopathologic significance in colorectal cancer (CRC) have not been determined yet. In the present study, one-step quantitative real-time polymerase chain reaction (qPCR) test and immunohistochemistry (IHC) analysis with CRC tissue microarrays (TMA) were employed to evaluate the mRNA and protein expression of SHIP2 in CRC. The results showed that SHIP2 expression in the mRNA and protein levels was significantly higher in CRC tissues than that in corresponding noncancerous tissues (both *P* < 0.05). The expression of SHIP2 protein in CRC was related to lymph node metastasis (*P* = 0.036), distant metastasis (*P* = 0.001), and overall survival (*P* = 0.009). Kaplan-Meier method and Cox multifactor analysis suggested that high SHIP2 protein level (*P* = 0.040) and positive distant metastasis (*P* = 0.048) were critically associated with the unfavorable survival of CRC patients. The findings suggested that SHIP2 may be identified as a useful prognostic marker in CRC and targeting CRC may provide novel strategy for CRC treatment.

## 1. Introduction

Colorectal cancer (CRC) is the third most common malignancy in the world, with an estimated incidence of more than 1.2 million new cases and over 600 000 deaths globally [1–3]. The number of patients who are suffering continues to rise, especially in most Asian countries [4]. During the last two decades, several countries including China, South Korea, and Japan have witnessed a two- to threefold rise in incidence of CRC. In China, for example, the total number of CRC cases increased by 19.1% and 17.7% in Chinese males and females from 2000 to 2005, respectively [5, 6]. Generally speaking, ulcerative colitis, familial adenomatous polyposis, and hereditary nonpolyposis colon cancer syndrome are the three major risks that contribute to the CRC development [7]. Despite the development of combined therapeutic modalities for CRC treatment, including surgical operation and combination of chemotherapy and adjuvant therapy, the overall prognosis of CRC remains unsatisfactory and the 5-year survival rate of CRC patients with metastasis is still under 10% [8, 9]. It is valuable and critical to discover molecular predictive markers for the prognosis, which would optimize the selection of therapeutic strategies and further improve patients' survival for CRC [10].

SH2-containing inositol 5′-phosphatase 2 (SHIP2) belongs to the phosphoinositol phosphatases family which plays important role in modulating signaling pathways relevant to both diabetes and cancer and generally regulates insulin signaling, cytoskeleton remodeling, and receptor endocytosis [11–13]. As one of the important lipid phosphatases that act downstream of phosphoinositide-3′ kinase (PI3K), SHIP2 converts phosphatidylinositol (3,4,5)-trisphosphate (PIP3) into phosphatidylinositol (3,4)-biphosphate (PIP2) and subsequently reduces the activation of PI3K/Akt signaling cascade, which exerts energetic function in tumor development and progression [14–16]. Under this circumstance, SHIP2 is supposed to show anticancer effects and several studies support this presumption [17, 18]. However, on the contrary, several studies elucidate the prooncogenic function of SHIP2 in certain type of cancer. For instance, high SHIP2 expression enhances cancer cell proliferation, while inhibition of SHIP2 suppresses cancer growth* in vitro* and distant metastases* in vivo *[12]. Similarly, SHIP2 performs oncogenically and high expression of SHIP2 indicates poor survival of breast cancer and laryngeal squamous cell carcinoma [19, 20]. SHIP2 also promotes cancer cell proliferation and metastasis by preventing epidermal growth factor receptor (EGFR) turnover and enhancing EGF-induced Akt activation [12, 21]. In light of the dual characteristics of SHIP2, the association of SHIP2 expression and cancer development still seems vague and little is known about the role of SHIP2 in CRC, especially the prognostic significance.

In this present paper, we detected SHIP2 expression in a number of CRC samples and analyzed the associations between SHIP2 expression and clinicopathologic items in CRC patients. Finally, the prognostic significance of SHIP2 was evaluated in 102 CRC cases.

## 2. Materials and Methods

### 2.1. Patients and Tissue Samples

A total of 15 fresh CRC tissues and matched tumor-adjacent noncancerous tissues were collected from the tissue bank of the Affiliated Hospital of Nantong University to perform quantitative real-time polymerase chain reaction (qPCR) test. 102 formalin-fixed, paraffin-embedded CRC tissues and corresponding tumor-adjacent normal tissues were also enrolled in this present study from the Affiliated Hospital of Nantong University from 2002 to 2005 to execute immunohistochemistry (IHC) analysis. Other pieces of clinicopathological information of 102 CRC cases, such as gender, age, tumor size, tumor location, histological type, tumor differentiation, serum CEA level, metastasis status, TNM stage, and overall survival, were collected from each patient's medical records simultaneously. Clinical staging was performed according to the latest revision of American Joint Committee on Cancer/International Union Against Cancer TNM staging system [22].

### 2.2. Detection of the SHIP2 Expression by One-Step qPCR Test

Total RNA was extracted from 15 cases of the frozen CRC tissues and the matched noncancerous tissues using the Trizol reagent (Invitrogen, Carlsbad, CA, USA) according to the manufacturer's guidelines. Total RNA extraction and one-step qPCR analysis were performed as previously described [23]. The primers for SHIP2 were as follows: forward primer 5′-TCG TCA CCA GCG ACC ATT C-3′ and reverse primer 5′-AGC CCT TTC TTG GAG ATG AAC TG-3′. The glyceraldehyde 3-phosphate dehydrogenase (GAPDH) mRNA level was used to standardize the measurements of SHIP2 and the primers for GAPDH were as follows: forward primer 5′-TGC ACC ACC AAC TGC TTA GC-3′ and reverse primer 3′-GGC ATG GAC TGT GGT CAT GAG-5′.

### 2.3. Detection of the SHIP2 Expression by IHC Analysis

Tissue microarray (TMA) was produced by Alenabio Biotech (Xi'an, China). Core tissue biopsies (2 mm in diameter) were collected from individual paraffin-embedded CRC sections and arranged in the recipient paraffin blocks. IHC analysis was performed as previously described [24]. Tissue sections were incubated with polyclonal goat anti-SHIP2 antibody (Santa Cruz Biotechnology, Santa Cruz, CA, USA) in TBS containing 1% bovine serum albumin for 1 hour. The secondary antibody used was horseradish peroxidase-conjugated anti-goat antibody (Dako Cytomation, Carpinteria, CA, USA).

SHIP2 immunostaining was scored by two independent pathologists according to intensity and percentage of positive cells simultaneously. Staining intensity was scored as follows: 0 (negative), 1 (weakly positive), 2 (moderately positive), and 3 (strongly positive). The percentage of SHIP2-positive cells was also scored according to four categories as follows: 1 was given for 0% to 10%, 2 for 11% to 50%, 3 for 51% to 80%, and 4 for 81% to 100%. The product of the intensity and percentage scores was used as the final staining score. The degree of SHIP2 staining was quantified using a two-level grading system as follows: samples with a sum score < 4 were considered as low SHIP2 expression while those with a sum score ≥ 4 were considered as high SHIP2 expression.

### 2.4. Statistical Analysis

Statistical analyses were performed by using SPSS 16.0 (SPSS Inc., Chicago, IL, USA) and STATA 12.0 (Stata Corporation, College Station, TX, USA). The expression of SHIP2 mRNA in fresh frozen CRC tissues and matched tumor-adjacent normal tissues normalized to GAPDH was analyzed with the Wilcoxon nonparametric signed-rank test. The relationship between SHIP2 protein expression and clinicopathological factors was evaluated by Chi-square test. Univariate and multivariate analyses were performed using Cox's proportional hazards regression models. Survival curves were calculated using the Kaplan-Meier method. For all tests, the significance level for statistical analysis was set at *P* < 0.05.

## 3. Results

### 3.1. Summarization of the Characteristics of 102 CRC Patients

A total of 102 CRC cases were enrolled from 63 men and 39 women in this present study. The mean age of all patients at the time of diagnosis was 63.08 ± 10.852 years. The tumor diameters of 44 cases were ≥5 cm and of 58 cases were <5 cm. The tumors of 87 cases were located in colon, 11 cases in rectum, and 4 cases in ileocecal junction. The histological type of tumor in 99 cases was adenocarcinoma, whereas the other 3 cases were identified as nonadenocarcinoma. Four patients suffered from a well-differentiated tumor, 82 had moderate tumor differentiation, and 7 had poor tumor differentiation. There were 11 cases with serum CEA level ≥ 15 ng/mL and 59 cases with serum CEA level < 15 ng/mL. Positive lymph node metastasis was noticed in 34 patients while distant metastasis was observed in 19 patients. According to TNM staging system, 63 patients were in stages I-II and 39 patients were in stages III-IV. Of all the 102 cases, 61 patients survived while 41 patients died.

### 3.2. Evaluation of SHIP2 mRNA Expression by qPCR Test

**Figure 1:**
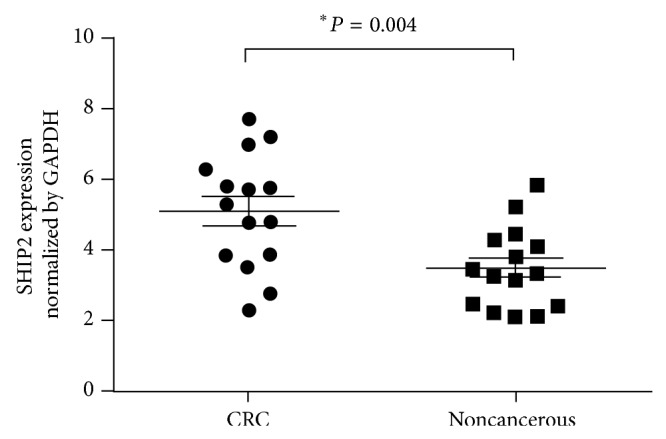
One-step quantitative real-time polymerase chain reaction (qPCR) test was employed to evaluate the expression of SHIP2 mRNA in colorectal cancer (CRC) and tumor-adjacent noncancerous tissues. When normalized to GAPDH, the expression of SHIP2 mRNA in CRC tissues (5.11 ± 0.419) was significantly higher than that in matched noncancerous tissues (3.48 ± 0.295) (*P* = 0.004).

Total RNA was extracted from 15 CRC tissues and matched noncancerous tissues and subsequently subjected to one-step qPCR for the evaluation of the expression of SHIP2 mRNA. When normalized to GAPDH, the means of SHIP2 mRNA expression in CRC tissues and the corresponding noncancerous tissues were 5.11 ± 0.419 and 3.48 ± 0.295, respectively (*t* = 3.174, *P* = 0.004). SHIP2 mRNA expression averaged 1.47-fold higher in CRC samples than that in noncancerous tissue samples (Figure 1).

### 3.3. Evaluation of SHIP2 Protein Expression by IHC Analysis

**Figure 2:**
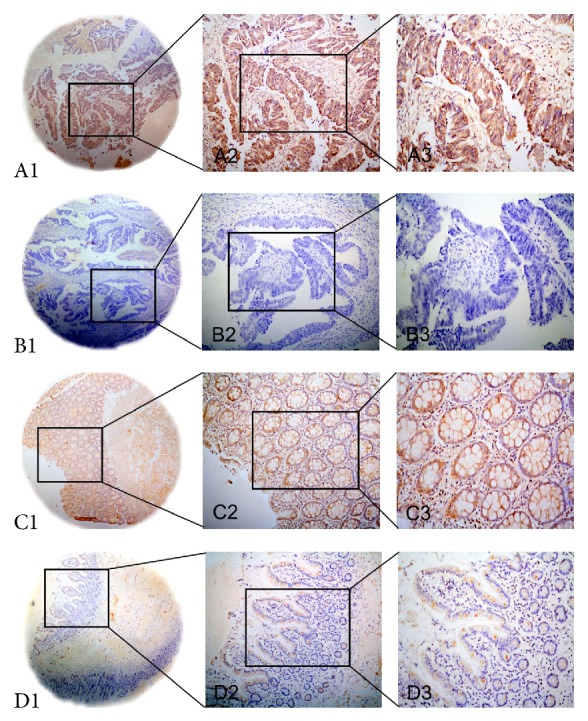
Representative images of SHIP2 protein expression in colorectal cancer (CRC) tissues and corresponding noncancerous tissues with tissue microarray (TMA). A1, A2, and A3: high immunohistochemical (IHC) staining of SHIP2 protein expression in CRC tissue samples. B1, B2, and B3: low IHC staining of SHIP2 protein expression in CRC tissue samples. C1, C2, and C3: high IHC staining of SHIP2 protein expression in noncancerous tissue samples. D1, D2, and D3: low IHC staining of SHIP2 protein expression in noncancerous tissue samples. Original magnification ×40 in A1, B1, C1, and D1; ×200 in A2, B2, C2, and D2; ×400 in A3, B3, C3, and D3.

IHC analysis was further performed to detect the SHIP2 protein expression in CRC. High SHIP2 expression was detected in 51 (50.0%) out of 102 CRC tissues and in 24 (23.5%) out of 102 tumor-adjacent noncancerous tissues. The results exhibited significant difference (*P* < 0.05) and were consistent with results of SHIP2 mRNA expression in qPCR test. Positive staining was mainly localized in the cytoplasm of CRC cells. Typical IHC staining patterns for SHIP2 in CRC are shown in Figure 2.

### 3.4. Relationships between SHIP2 Protein Expression and Clinicopathological Items

The relationships between high SHIP2 protein expression and selected clinicopathological items were shown in Table 1. High SHIP2 protein expression was significantly related to lymph node metastasis (*P* = 0.036), distant metastasis (*P* = 0.001), and overall survival (*P* = 0.009) (Table 1).

### 3.5. Survival Analysis

**Figure 3:**
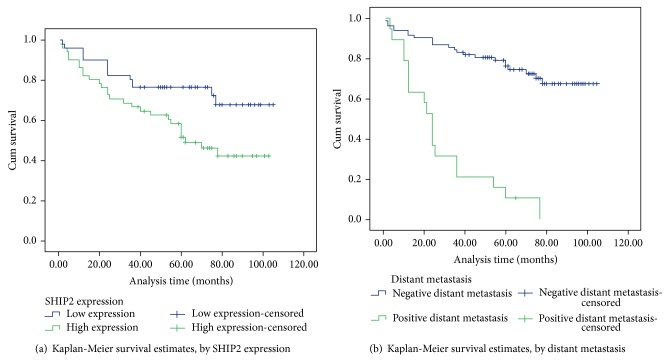
Survival analysis of colorectal cancer (CRC) patients by Kaplan-Meier method. (a) Overall survival rate in patients with high SHIP2 protein expression (green line) was significantly lower than that in patients with low SHIP2 expression (blue line). (b) Overall survival rate in patients with positive distant metastasis (green line) was significantly lower than that in patients with negative distant metastasis (blue line).

In order to evaluate the prognostic role of SHIP2, survival analyses were subsequently executed. In univariate analysis, 4 items including high SHIP2 protein expression (*P* = 0.017), lymph node metastasis (*P* = 0.001), distant metastasis (*P* = 0.001), and TNM stage (*P* = 0.001) showed a significant correlation with the overall survival rate of 102 CRC patients. Furthermore, multivariate analysis confirmed that high SHIP2 expression (*P* = 0.040) and distant metastasis (*P* = 0.001) were two independent prognostic factors for CRC (Table 2). Kaplan-Meier survival curves demonstrated that CRC patients with high SHIP2 expression and positive distant metastasis encountered a significantly poorer survival time (Figure 3).

## 4. Discussion

In insulin signaling, SHIP2 plays a substantial role in the negative regulation of insulin sensitivity [14]. SHIP2 hydrolyzes the PI3K product PIP3 to PIP2 and inhibits the performance of PI3K/Akt signaling simultaneously [25]. In turn, SHIP2 suppresses cell growth through regulating intracellular insulin sensitivity. On the other hand, SHIP2 also regulates cell adhesion pathways which take great effects in the movement of cells. Considering the fact that the SHIP2 may function diversely in adhesion signaling by comparing to its role in insulin signaling, it is highly possible that abnormal expression of SHIP2 in some pathological conditions may impart its capability on cancer development from insulin regulation in normal physiological situations [12]. Accordingly, a review by Suwa et al. states that SHIP2 shows both prooncogenic effects and anticancer effects for SHIP2 function may differ based on interactive and imbalanced modulation by upstream stimulators including insulin, EGF, and IGF-I and downstream molecules including Akt2, tyrosine kinase receptor, and focal adhesion adaptor proteins [26]. For CRC, a great number of studies pointed the crucial relationships in the expression of adhesion proteins and catenin pathway with CRC development [27–29]; the exact role of SHIP2 in CRC is barely explored.

In this study, the qPCR result showed that the SHIP2 mRNA expressions in CRC tissues were higher than those in normal noncancerous tissues. Furthermore, TMA with CRC samples was constructed and IHC analysis was performed to confirm that higher SHIP2 protein expression was detected in CRC samples than that in matched noncancerous samples. The data elucidated the oncogenic role of SHIP2 in tumorigenesis in CRC and were consistent with previous studies that indicated high expression of SHIP2 in cancer tissues [19, 20]. Besides, high SHIP2 expression in CRC was correlated with several clinical attributes, including lymph node metastasis, distant metastasis, and overall survival. The results showing the prooncogenic effect of SHIP2 were in accord with our latest study concerning SHIP2 expression in lung cancer [23]. Similarly, a number of mechanical speculations have been raised to explore the characteristics of SHIP2 in cancer development. SHIP2 may regulate cell spreading and induce generation of local pools of PIP2, which play critical parts in the actin remodeling involved in the movement of cells [30]. SHIP2 may perform as a scaffolding protein to promote specific interaction with substantial cytoskeleton regulators, thereby subsequently controlling actin remodeling [26, 31]. To summarize, SHIP2 is believed to modulate the ability of cells to migrate and attach via maintenance and remodeling of the actin cytoskeleton. Hence the loss of focal adhesion leading to inhibitory spreading ability indicates defective cell locomotion, which in turn regulates cancer cell migration and invasion during metastasis. Our data of the associations between SHIP2 expression and clinicopathologic items in CRC in this present study were also in agreement with the above reports concerning the prooncogenic effects of SHIP2.

In addition, high SHIP2 protein expression, lymph node metastasis, distant metastasis, and TNM stage were revealed to be correlated with overall survival of CRC patients by univariate analysis while high SHIP2 expression and distant metastasis were further authenticated as independent predictors for unfavorable overall survival of CRC by multivariate analysis. Kaplan-Meier analysis finally demonstrated that the life span of patients with high SHIP2 expression was longer than that of patients with negative expression. Several studies, including two researches of ourselves, support our data announcing the prognostic role of SHIP2 in cancer [19, 20, 23, 24]. Hence, it is rational to presume that SHIP2 plays oncogenic role dominantly in CRC based on this present research. Detailed studies of PI3K/Akt signaling and adhesion protein interacting with SHIP2 in various cell types or tissues are necessary to clarify the potential role of SHIP2 in procarcinogenesis.

In conclusion, we identified for the first time that a higher expression of SHIP2 in CRC tissues compared to the noncancerous tissues and SHIP2 might play an essential role as a prognostic marker of survival in patients with CRC. Our study is helpful in understanding the characteristics of SHIP2 in CRC development.

## Figures and Tables

**Table 1:** The relationship between SHIP2 expression and clinical attributes of 102 CRC patients.

Groups	Number	SHIP2		*χ* ^2^	*P* value
		+	%		
Gender					
Male	63	31	49.2	0.04	0.839
Female	39	20	51.3		
Age (years)					
≥60	68	34	50.0	0.00	1.000
<60	34	17	50.0		
Tumor size (cm)					
≥5	44	21	47.7	0.16	0.689
<5	58	30	51.7		
Tumor location					
Colon	87	43	49.4	1.10	0.576
Rectum	11	5	45.5		
Ileocecal junction	4	3	75.0		
Histological type					
Adenocarcinoma	99	48	48.5	3.09	0.079
Nonadenocarcinoma	3	3	100.0		
Tumor differentiation					
Well	4	1	25.0	1.32	0.518
Moderately	82	44	53.7		
Poorly	7	4	57.1		
Insufficient data	9	2			
Serum CEA level (ng/mL)					
≥15	11	6	54.5	0.30	0.581
<15	59	25	42.4		
Insufficient data	32	20			
Lymph node metastasis					
Positive	34	22	64.7	4.41	0.036^*^
Negative	68	29	42.6		
Distant metastasis					
Positive	19	17	89.5	14.55	0.001^*^
Negative	83	34	41.0		
TNM stage					
Stages I, II	63	27	42.9	3.36	0.067
Stages III, IV	39	24	61.5		
Overall survival					
Survival	61	24	39.3	6.89	0.009^*^
Death	41	27	65.9		

^*^ *P* < 0.05.

**Table 2:** Univariate and multivariate analyses of prognostic factors in 102 CRC patients for overall survival.

	Univariate analysis			Multivariate analysis		
	HR	*P* > |*z*|	95% CI	HR	*P* > |*z*|	95% CI
SHIP2 expression						
High versus low	2.20	0.017^*^	1.151–4.189	2.05	0.040^*^	1.031–3.820
Gender						
Male versus female	1.44	0.281	0.744–2.774			
Age (years)						
≥60 versus <60	0.83	0.562	0.439–1.565			
Tumor size (cm)						
≥5 versus <5	1.29	0.410	0.701–2.388			
Tumor location						
Colon versus rectum versus ileocecal junction	0.68	0.354	0.306–1.528			
Histological type						
Adenocarcinoma versus nonadenocarcinoma	1.05	0.960	0.145–7.662			
Tumor differentiation						
Well and moderately versus poorly	1.50	0.413	0.569–3.943			
Serum CEA level (ng/mL)						
≥15 versus <15	2.33	0.057	0.976–5.573			
Lymph node metastasis						
Positive versus negative	2.91	0.001^*^	1.573–5.408	1.26	0.668	0.443–3.568
Distant metastasis						
Positive versus negative	7.06	0.001^*^	3.687–13.521	5.09	0.001^*^	2.034–12.736
TNM stage						
Stages I-II versus stages III-IV	0.25	0.001^*^	0.129–0.466	0.72	0.638	0.188–2.789

^*^ *P* < 0.05.
